# Preoperative estimation of retinal hole location using ultra-wide-field imaging

**DOI:** 10.1080/07853890.2023.2258790

**Published:** 2023-09-19

**Authors:** Donghui Li, Nalei Zhou, Rui Gao, Jialiang Duan, Qingli Shang

**Affiliations:** Ophthalmology Department, The Second Hospital of Hebei Medical University, Shijiazhuang, China

**Keywords:** Ultra-wide-field imaging, rhegmatogenous retinal detachment, scleral buckling, retinal hole

## Abstract

**Background/Objective:**

Accurate localization of retinal holes is essential for successful scleral buckling (SB) surgery. We aimed to verify the feasibility of using ultra-wide-field (UWF) imaging for preoperative estimation of retinal hole location.

**Patients and Methods:**

We observed 21 eyes from 21 patients with rhegmatogenous retinal detachment (RRD) who underwent successful SB. They were treated at the Department of Ophthalmology of the Second Hospital of Hebei Medical University between November 2020 and November 2021. UWF fundus photography using an Optos device was performed at different steering positions 1 day before, 1 day after, and 1 month after SB. Using the preoperative fundus images, we measured the transverse diameter of the optic disc (D1) and the distance from the centre of the retinal holes to the ora serrata (D2). The accurate transverse diameter of the optic disc (Dd) was measured preoperatively using optical coherence tomography. The same surgeon measured the scleral chord lengths intraoperatively from the limbus to the located retinal hole marked on the sclera using an ophthalmic calliper. Statistical software was used to analyze the consistency of scleral chord length between the retinal hole and the limbus, which was estimated by preoperative UWF imaging and was measured using an ophthalmic calliper intraoperatively.

**Results:**

There was no statistically significant difference in the scleral chord length between the retinal holes and the limbus, which was estimated by preoperative UWF fundus photography and was measured by the calliper during surgery.

**Conclusion:**

It is feasible to locate retinal holes using UWF fundus photography before SB, which is helpful for quick localization, thereby reducing the learning curve of SB surgery.

## KEY MESSAGES

Preoperative ultra-wide-field imaging can provide abundant information about retinal holes and is helpful for assessing their location before surgery.

In this prospective cohort study of 21 patients, 25 retinal holes in four quadrants were observed.

Axial length and the position of the holes have little impact on preoperative ultra-wide field imaging assessment.

## Introduction

Rhegmatogenous retinal detachment (RRD) is the most common type of retinal detachment; blindness may occur in the affected eye unless surgery is performed. The primary surgical methods include scleral buckling (SB), pars plana vitrectomy (PPV), and pneumatic retinopexy (PR) [1]. SB is a traditional technique for RRD and can be used as a major surgical method with a high success rate and few complications. SB can also be used as an auxiliary treatment for vitrectomy. In many cases, SB is an effective treatment for RRD, especially for RRD secondary to retinal lattice degeneration with atrophic retinal holes [2]. SB also has a lower incidence of iatrogenic breaks and secondary cataracts than PPV [3]. Despite this, SB causes less disturbance to the vitreous body [4]. However, in recent years, the use of SB in clinical practice has been declining, and it is largely being replaced by PPV [5,6]. Therefore, some opinions state that vitrectomy should be performed more carefully, and SB should be reassessed [7]. The reasons for the decrease in clinical SB use may be multifaceted; the rapid development of vitrectomy instruments and techniques may be the main reason, but the learning difficulty of SB surgery may also cause it.

Accurate localization of retinal holes during surgery determines the success of SB. Currently, most intraoperative localization methods require an indirect ophthalmoscope to observe the hole. The difficulty in mastering this technique is an important reason for the long learning curve of SB. New SB surgery, which is assisted by chandelier endoillumination, makes it easier for surgeons to observe the fundus and locate holes, but it also requires additional scleral incisions and instruments [8]. Although traditional surgery requires surgical experience and skill, it is still necessary for clinicians to master the procedure because its long-term safety profile has been proven.

The Optos ultra-wide-field (UWF) imaging system is the latest generation of fundus examination instruments with non-contact and ultra-wide-field characteristics [9]. Surgeons can obtain abundant information about the retina, including data on the number, shape, and approximate position of retinal holes, using preoperative UWF imaging. However, there are few reports on the accurate localization of retinal holes using UWF imaging [10]. The present work aimed to study the feasibility of locating retinal holes using UWF fundus photography before surgery. We expect this to assist surgeons in the localization of retinal holes and shorten the learning curve of SB.

## Patients and methods

This prospective cohort study included patients diagnosed with RRD and treated with SB in the Department of Ophthalmology of the Second Hospital of Hebei Medical University between November 2020 and November 2021. Patients with retinal holes detected by UWF fundus photography were included. The following patients were excluded: those who had undergone or needed cataract surgery and/or vitrectomy; those with PVR C2 or above, giant retinal tears, or dialysis of the ora serrata; and those with RRD where holes could be detected using traditional methods (binocular indirect ophthalmoscope and a three-mirror lens) but not by UWF images. A total of 21 eyes from 21 patients were included. This study adhered to the tenets of the Declaration of Helsinki, and the protocols were approved by the Ethical Committee of The Second Hospital of Hebei Medical University. Written informed consent was obtained from the patients enrolled in the study.

### Data collection and analysis

#### Preoperative assessment

Ultra-wide-angle fundus images were taken using an Optos Ultra-Widefield Fundus Camera (Optos, Dunfermline, UK, 200Tx). These images were taken 1 day preoperatively, 1 week postoperatively, and 1 month postoperatively. Pupils were dilated to the greatest extent before taking images. The images of the central, superior, inferior, nasal, and temporal retina of each eye were taken by the same experienced technician. During the process, patients’ eyelids were lifted by the technician, and they had to try their best to stare in the corresponding directions. Images of the retinal holes were taken in a similar manner. All images were taken when the ‘green in-focus’ signal was obtained on the Optos machine.

The images of the inner surface of the eye, which is nearly spherical, cannot be mapped to a flat surface without distortion. The size and shape of some structures can be greatly distorted with further eccentricity in taking images. Therefore, we standardized the images to make them more accurate. We exported images from the Optos and converted them to grayscale in PhotoshopCS3 (Adobe, Inc., San Jose, CA). Images centred on the posterior portion of the eye were inspected for sharpness and lack of systemic distortion across the image. We selected the best image and considered it to be the best image of the periphery. To standardize the resultant images, histogram stretching was performed. Peripheral images were transformed using elastic deformation, and the posterior portions of peripheral images matched the base reference images. Warped images were merged into a montage [11]. The UWF imaging range can reach the ora serrata through eye position guidance. Moreover, pupillary dilation and eyelid lifting can enlarge the imaging range [12]. Therefore, we assumed that the ora serrata corresponded to the blood-free zone at the edge of the obtained image when the eye turned to the limit range.

**Figure 1.**
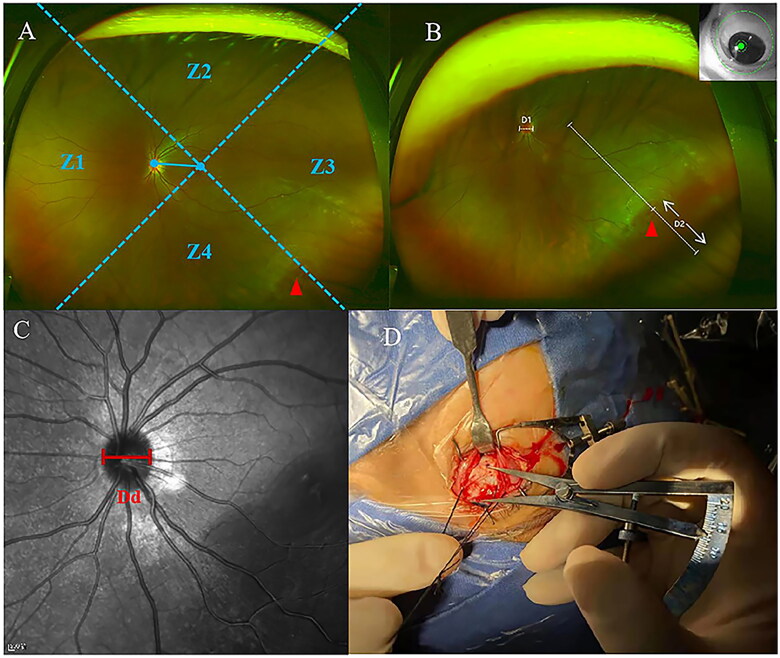
(A) Z1, the nasal side; Z2, the superior side; Z3, the temporal side; Z4, the inferior side. (B) D1, optic disc transverse diameter; D2, the distance between the centre of the retinal hole and the edge of the image in the extension line of the macular fovea and retinal hole. The Optos images were taken when the patients did their best to stare in the direction of the retinal holes. (C) Dd, the optic disc transverse diameter measured by optical coherence tomography. (D) The chord length is measured between the limbus and retinal hole mark during the operation. Red triangles: retinal holes.

The macular centre was considered the centre, and the horizontal position was determined through the connection between the macular centre and the optic disc centre. Based on the location of the hole, the combined standardized fundus image was divided into four regions: the nasal side (Z1), the superior side (Z2), the temporal side (Z3), and the inferior side (Z4) (Figure 1A). We measured the optic disc transverse diameter (D1) using the combined tandardized images. The line between the macular fovea and the centre of the retinal hole was made and extended to the edge of the image. The distance between the centre of the retinal hole and the edge of the extension line was D2 (Figure 1B). The standardization of the images and the data measurement were finished independently by two researchers (Li and Shang). Data with large differences were measured again (Gao). The mean was used in the next calculation.

The optic disc transverse diameter was measured by an optical coherence tomography scanner (Heidelberg Engineering, Heidelberg, Germany). Automatic software with automated measurement was used to determine the transverse diameter of the optic disc of each eye. We used the transverse diameter values of the optic disc (Figure 1C). A Zeiss IOL Master laser interferometer (Optical Biometry, IOL Master; Carl Zeiss Meditec AG, Jena, Germany) was used to measure the axial length (AL).

**Figure 2.**
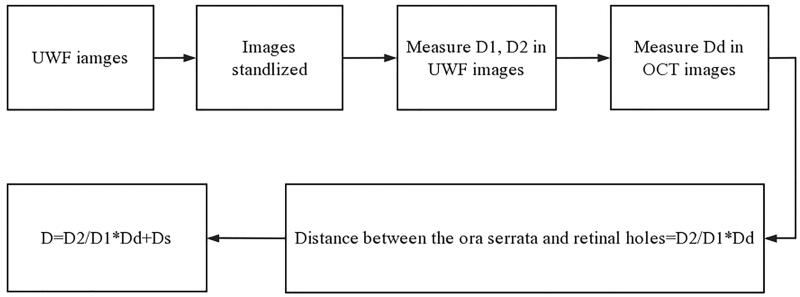
Flowchart of computational formula. D1, optic disc transverse diameter. D2, the distance between the centre of the retinal hole and the edge of the image in the extension line of the macular fovea and retinal hole. Dd, the optic disc transverse diameter measured by optical coherence tomography. Ds, distance between the ora serrata and limbus.

Based on the measured AL, patients were divided into two groups: AL ≥ 26 mm and AL < 26 mm. The optic disc transverse diameter measured using optical coherence tomography was labelled Dd. The preoperative scleral chord length between the limbus and retinal hole was calculated as follows: D = D2/D1 * Dd + Ds (where Ds = distance between the ora serrata and limbus). Straatsma et al. measured the distance from the serrated margin to the Schwalbe’s line from the eye of a cadaver as 6.14 ± 0.85 mm superior, 6.20 ± 0.76 mm inferior, 5.73 ± 0.81 mm on the nasal side, and 6.53 ± 0.75 mm on the temporal side [13]. However, the chord length from the limbus to the hole was actually measured from the outside of the eyeball during the operation. The distances between the ora serrata and the limbus used in the calculation were according to personal experience. To simplify the calculation, we added 7 mm to the nasal side, 8 mm to the temporal side, and 7.5 mm to the superior and inferior sides. The preoperative estimation process is shown in a flowchart in Figure 2.

#### Intraoperative measurement

Locating and marking of retinal holes on the sclera were performed by the same experienced surgeon (Duan). The statistics calculated from the images were not shared with the surgeon who performed the surgery. After locating the hole in the surgery, an ophthalmic calliper was used to measure the scleral chord length between the limbus and scleral markers of the retinal holes (Figure 1D). Holes were divided into four regions: the superior, inferior, nasal, and temporal. The region and scleral chord length of each hole were recorded.

#### Statistical analyses

The preoperative estimated scleral chord length was calculated and compared with the measured scleral chord length during surgery. All analyses were performed using SPSS statistical software (v21; IBM Corp., Armonk, NY, USA). Continuous, normally distributed data were expressed as mean ± standard deviation and were compared using a paired t-test. *p* values < 0.01 were considered statistically significant. Calculation of preoperative scleral chord length and statistical analysis were completed by another researcher (Zhou) who did not participate in data collection.

## Results

### Patient demographics

The data and images of 21 eyes of 21 patients (9 males and 12 females) were collected. The average age of the patients was 27.1 ± 8.6 (range: 16–53) years; 25 retinal holes were observed. The average AL of all eyes was 26.32 ± 1.30 (range: 24.29–29.23) mm. The mean preoperative best-corrected visual acuity (BCVA) was 0.36 ± 0.35 LogMAR, ranging from 1.4 to −0.1 LogMAR. All included eyes were phakic (Table 1).

**Table 1.** Patients’ characteristics.

Case number	AL (mm)	Location of retinal holes	Estimated chord length (mm)	Actual chord length (mm)
1	28.72	Z4	12.51	13.5
2	25.52	Z4	13.99	15
3	24.87	Z3	16.66	15
4	26.75	Z3	15.77	15.5
5	25.94	Z3	15.02	15
6	24.74	Z2	15.15	15
7	26.42	Z4	13	12
8	24.59	Z4	13.49	15
9	26.46	Z1	16.24	15
10	25.21	Z3	11.11	10
11	29.23	Z4	12.74	12
12	24.29	Z4	12.71	12
13	25.71	Z2	13.42	14
14	27.06	Z2 Z3	13.54 14.24	14 14.5
15	26.77	Z4	13.14	14
16	26.59	Z3	13.28	12
17	27.21	Z2 Z2 Z3	14.83 14.53 14.79	14.5 14.5 15
18	26.47	Z3 Z2	14.85 14.56	14.5 14.5
19	26.54	Z2	12.17	12
20	25.55	Z4	14.54	14
21	28.00	Z4	13.47	14

Abbreviations: AL: axial length.

### Determination of retinal hole location based on intra- and postoperative Optos imaging

There were 16 holes in the AL ≥ 26 mm group. The average estimated preoperative scleral chord length was 13.99 ± 1.18 mm, and the average actual intraoperative chord length was 13.84 ± 1.19 mm; the difference between them was 0.14 (range: −0.99–1.28) mm and not significant (*p* = 0.445 > 0.01). There were 9 holes in the AL < 26 mm group. The average estimated preoperative scleral chord length was 14.01 ± 1.59 mm, and the average actual intraoperative chord length was 13.89 ± 1.76 mm; the difference between them was 0.12 (range: −1.51–1.66) mm and not significant (*p* = 0.731 > 0.01). Comparing the partitions, there was no statistically significant difference between the preoperative and intraoperative estimated chord lengths in the Z2 (*p* = 0.080 > 0.01), Z3 (*p* = 0.751 > 0.01), and Z4 (*p* = 0.522 > 0.01) regions. There was only one case that involved the Z1 region, which was Case 9. The estimated preoperative chord length was 16.24 mm, and the actual intraoperative measurement was 15 mm. This data was too small to be analyzed.

## Discussion

RRD is a common ophthalmic disease. SB is a traditional technique for retinal detachment repair, and long-term studies have reported a success rate of 95% [14]. However, recently, owing to the development and popularization of vitrectomy, the use of SB has reduced gradually. A study in the United States showed that in RRD surgery, PPV use increased by 72%. SB without vitrectomy decreased by 69% from 1997 to 2007 [4]. Another study investigated 2000–2014 data and reported that the number of RRD cases in which vitrectomy was performed increased from 13,814 to 19,288, and the number of cases in which SB was performed decreased significantly from 6502 to 1260 [5]. Regarding retinal detachment repair surgery, vitrectomy, SB, and PR were performed in 83%, 5%, and 12% of cases, respectively [5]. However, compared with vitrectomy, SB is suitable for young patients with phakia and especially suitable for RRD secondary to retinal lattice degeneration with atrophic retinal holes [15]. Another advantage of SB is that there are few complications such as cataracts, preretinal proliferation, recurrent traction retinal detachment, and new operation-related holes [6].

The key to successful SB is the closure of the retinal holes, and the key to the closure of the retinal holes is accurate localization. Therefore, accurate location determination of retinal holes during surgery decides the success of SB. Presently, the localization methods of retinal holes are divided into preoperative and intraoperative methods. Preoperatively, surgeons examine the fundus using a binocular indirect ophthalmoscope and a three-mirror lens to detect all possible holes. The location of the holes and the distance between the holes and the limbus are estimated simultaneously. This approach is not quantitative and requires individual experience. Intraoperatively, pressing the sclera and observing by the indirect ophthalmoscope is the most common localization method [16]. However, skill comes from practicing the surgery, as SB has a lengthy learning curve.

There have been recent advances in SB to make the learning process easier. SB assisted by 25-G fibre, and chandelier endoillumination has many advantages over traditional methods. For instance, it is easier for surgeons to observe the fundus and even the peripheral retina under a microscope to detect small retinal holes which are difficult to identify with indirect ophthalmoscopy [8]. However, the surgery needs the sclera incision to be extended to the vitreous cavity, which may alter the physiological environment of the vitreous. The risk of postoperative endophthalmitis and other complications, such as retinal holes secondary to vitreous incarceration at the insertion point of the cannula, can be increased [17,18]. In addition, not all clinics are equipped with chandelier endoillumination.

Ultra-wide-angle imaging has greatly enhanced our realization of the peripheral retina preoperatively. A 200° range of the fundus can be observed in one picture. With the patient’s cooperation, the range can be extended to the ora serrata by guiding the eye position (upper, lower, nasal, and temporal directions) [19]. Information regarding retinal holes can be observed preoperatively with UWF imaging, but there is little research analyzing this information quantitatively [10]. We designed this research to locate the retinal hole preoperationally using this technique.

In our research, 21 patients were included, and 25 retinal holes were observed. The inner surface of the eye is a three-dimensional sphere, which is difficult to map to a two-dimensional plane without distortion. Deviations measured directly from the wide-angle image and the actual data of the eyeball are uncertain [11]. Therefore, we standardized the images and used the ratio between the transverse diameter of the optic disc and the distance from the measured hole to the edge of the ora serrata to reduce the error caused by direct measurement. Meanwhile, we divided the data from AL and location again to reduce error.

From our results, the average difference between the estimated chord length of retinal tears and the actual measured chord length was 0.13 (range: −1.51–1.66) mm. There was no statistically significant difference between the estimated chord length and the actual measured chord length in the AL ≥ 26 mm group (*p* =   0.445 > 0.01) and the AL < 26 mm group (*p*  = 0.731 > 0.01). Regarding the different zones of the holes, statistical analyses based on the location of the retinal holes showed that there was no statistically significant difference in the retinal tears in the Z2, Z3, or Z4 regions. However, only one case involved the Z1 region, and the data was insufficient for analysis. We also observed on the postoperative images that all the retinal holes were on the buckle, suggesting the feasibility of locating retinal holes using UWF photography preoperatively.

In clinical practice, we used a relatively simple method of calculation. We used the Optos software to acquire the optic disc transverse diameter (D1) and the distance between the centre of the retinal hole and the ora serrata (D2). The optic disc diameter was estimated to be 1.5 mm. We then used the formula D = D2/D1 * 1.5+ Ds, with *Ds* being the distance between the ora serrata and limbus. We added 7 mm to the nasal side, 8 mm to the temporal side, and 7.5 mm each to the superior and inferior sides. Supplementary Materials 1 and 2 present the detailed data and our analysis. Interestingly, there were also no statistically significant differences between these simplified preoperative estimate methods and intraoperative estimated chord lengths (*p* = 0.119 > 0.01). According to resident training in our study site, our method improves the learning curve of localization of retinal holes skill in SB surgery.

There are few studies on calculations of the retinal hole positions using a UWF image. Ishikawa et al. calculated the distance between the posterior edge of the scleral buckle and cornea on a UWF image after SB and compared it to the distance measured during surgery. A regression equation was obtained based on the data. The position of the retinal hole in the preoperative image was brought into the regression equation, and the results fell within the range of the scleral buckle [10]. Their study revealed the feasibility of estimating the intraocular distance using UWF imaging. However, the shape of the eyeball, which could not be estimated accurately, was changed in the area where the buckle was performed. The change in the wide-angle image could not be predicted accurately; this is an unavoidable error when calculating using postoperative images. In our study, preoperative UWF images were used to avoid this problem; this is more consistent with the real therapeutic process.

However, our study has some limitations. First, the error caused by the distortion of the wide-angle image could not be completely avoided. Single-shot imaging of the Optos cannot cover the entire retina. We obtained images of the peripheral retina by guiding the direction of the patient’s gaze. Therefore, even a small deviation may significantly impact the final plane image. Second, the chord length from the ora serrata to the limbus that we used in our study (8 mm, 7 mm) was based on estimation and may have individual variations. Third, our data was insufficient for nasal position analysis. Last, the patients in our study might not be representative of all RRD populations; thus, generalizability based on different phakic statuses, large retinal tears, and bullous retinal detachment may be limited, which warrants future investigation.

## Conclusions

There was no statistically significant difference in the scleral chord length between the retinal holes and the limbus estimated by UWF fundus photography used preoperatively and measured intraoperatively. Preoperative analysis using UWF imaging helps surgeons estimate the scleral chord length between retinal holes and the limbus. Thereby, it may help to improve the learning process for SB surgery.

## Supplementary Material

Supplemental Material

## Data Availability

All data supporting the findings of this study are available from the corresponding author upon request.
