# Supplementary material for: Preoperative estimation of retinal hole location using ultra-wide-field imaging
Source: Ann Med. 2023 Sep 19;55(2):2258790. doi: 10.1080/07853890.2023.2258790 (PMC10512843; doi:10.1080/07853890.2023.2258790)
Supplement: Supplemental Material [file IANN_A_2258790_SM5928.zip › Supplementary Material 1.docx]

| Case number | Location of retinal holes | Estimated chord length (mm) | Actual chord length (mm) |
| --- | --- | --- | --- |
| 1 | Z4 | 12.04 | 13.5 |
| 2 | Z4 | 14.64 | 15 |
| 3 | Z3 | 13.91 | 15 |
| 4 | Z3 | 17.31 | 15.5 |
| 5 | Z3 | 14.66 | 15 |
| 6 | Z2 | 14.39 | 15 |
| 7 | Z4 | 11.60 | 12 |
| 8 | Z4 | 14.29 | 15 |
| 9 | Z1 | 15.02 | 15 |
| 10 | Z3 | 10.72 | 10 |
| 11 | Z4 | 11.41 | 12 |
| 12 | Z4 | 11.84 | 12 |
| 13 | Z2 | 13.04 | 14 |
| 14 | Z2、Z3 | 13.50、13.70 | 14、14.5 |
| 15 | Z4 | 13.08 | 14 |
| 16 | Z3 | 13.64 | 12 |
| 17 | Z2、Z2、Z3 | 14.02、13.77、14.54 | 14.5、14.5、15 |
| 18 | Z3、Z2 | 14.86、14.98 | 14.5、14.5 |
| 19 | Z2 | 12.59 | 12 |
| 20 | Z4 | 13.39 | 14 |
| 21 | Z4 | 13.11 | 14 |

Table 1: Patients’ characteristics

| Number of retinal holes | Estimated chord length (mm) | Actual chord length (mm) | P value* |
| --- | --- | --- | --- |
| 25 | 13.60 ± 1.42 | 13.86 ± 1.39 | 0.119 |

Table 2: Date analyze of simplified estimate in clinical.
