# Supplementary material for: Preoperative estimation of retinal hole location using ultra-wide-field imaging
Source: Ann Med. 2023 Sep 19;55(2):2258790. doi: 10.1080/07853890.2023.2258790 (PMC10512843; doi:10.1080/07853890.2023.2258790)
Supplement: Supplemental Material [file IANN_A_2258790_SM5928.zip › Supplementary Material 2.docx]

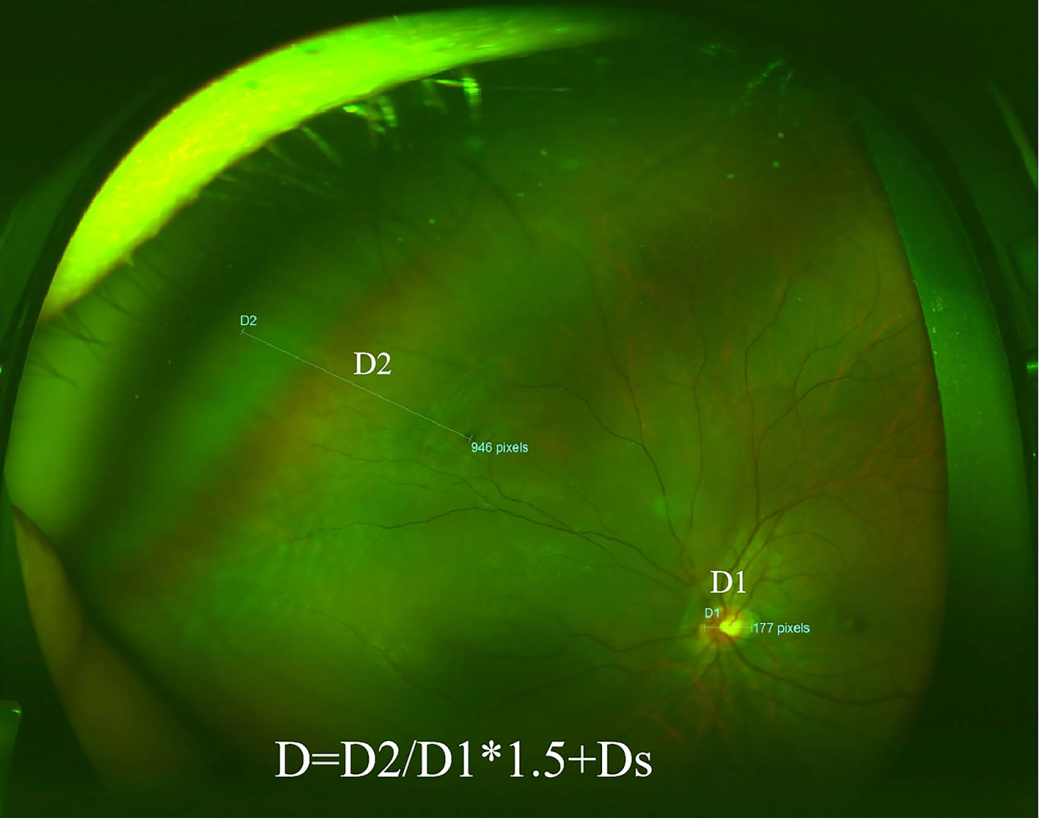


Supplement Material 2: Actual calculation in clinical practice. D1, optic disc transverse diameter; D2, distance between the center of the retinal hole and the edge of the image in the extension line of the macular fovea and retinal hole. Ds, distance between the ora serrata and limbus. Added 7 mm to the nasal side, 8 mm to the temporal side, and 7.5 mm to the superior and inferior sides. In this patient, D=D2/D1*1.5+Ds=946/177*1.5+7mm (the nasal side)=15.02 mm. And the actual chord length is 15 mm.
